# Supplementary material for: Switching from Sugar- to Artificially-Sweetened Beverages: A 12-Week Trial
Source: Nutrients. 2023 May 4;15(9):2191. doi: 10.3390/nu15092191 (PMC10181485; doi:10.3390/nu15092191)
Supplement: Supplementary file 1 [file nutrients-15-02191-s001.zip › nutrients-2341964-supplementary.pdf]

Supplementary information

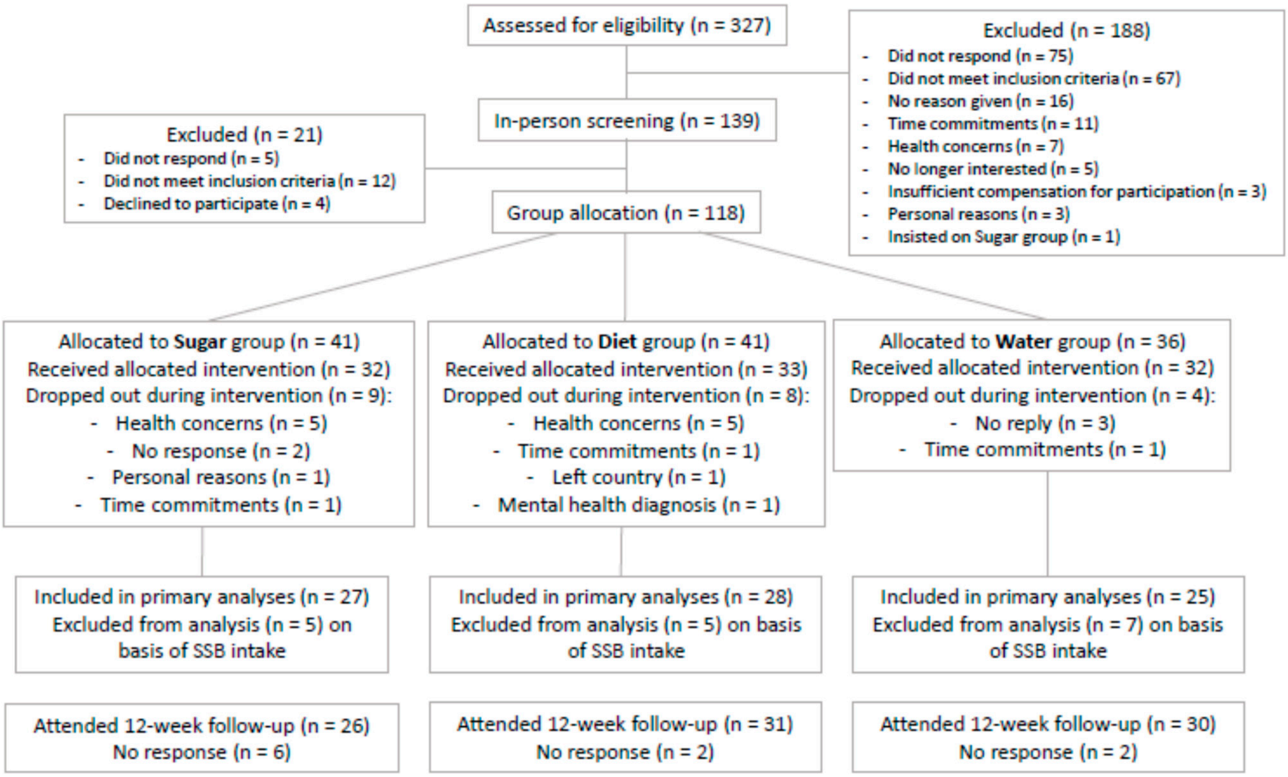

Supplementary Figure S1. Participant recruitment and attrition. Of the 118 eligible participants were allocated to the 3 groups, 97 completed the 12-week intervention. Seventeen participants were identified as outliers on measures of self-reported SSB intake during the intervention and were excluded from analyses.

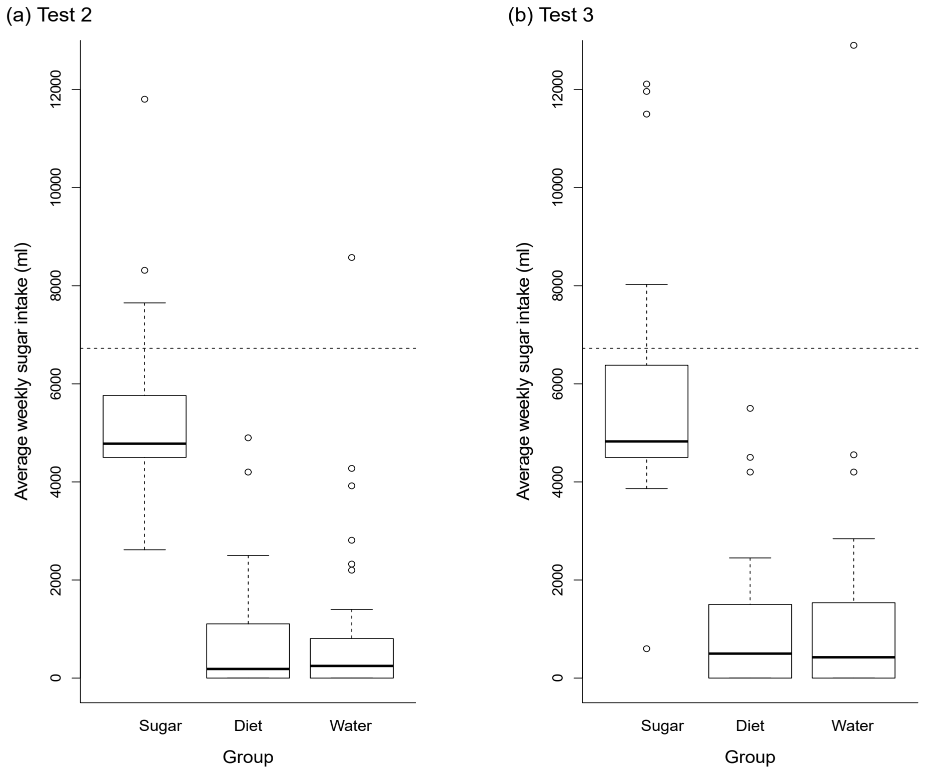

Supplementary Figure S2. Box and whisker plot of average SSB intake (ml/week) at Test 2 ((a), weeks 1-6) and Test 3 ((b), weeks 7-12). The dotted line depicts average baseline intake for all participants, which did not differ between groups. Seventeen participants were identified as outliers (SSB intake > 1.5 x interquartile range for the group) and were excluded from analyses.

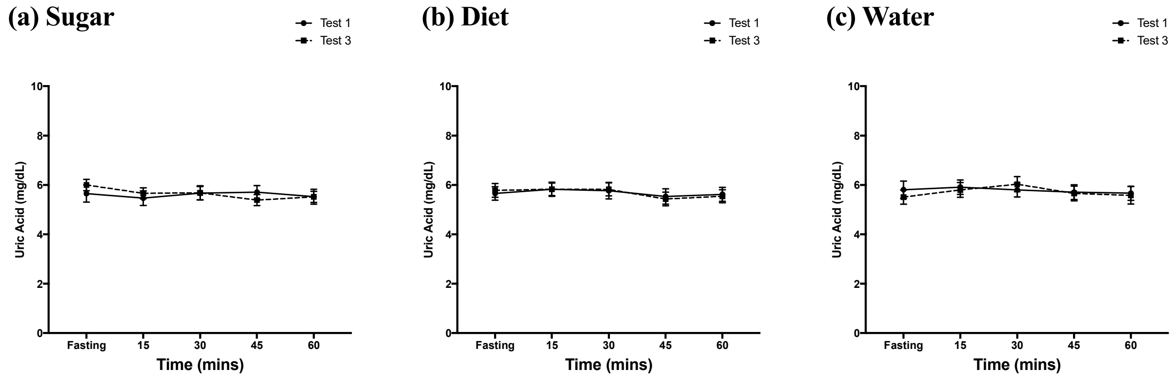

Supplementary Figure S3. Uric acid in blood was measured in a subset of participants in Sugar ((a), n = 18), Diet ((b), n = 15) and Water ((c), n = 13) concomitantly with glucose measures during the OGTT held at Tests 1 and 3. Repeated measures ANCOVA with Group and Sex (between-subjects), and Test and Time (within-subjects) as factors revealed a marginally significant main effect of Time, $F(4, 148) = 2.47$, $p = .048$, $\eta p2 = .062$, and a significant interaction between Time and Group, $F(8,148) = 2.16$, $p = .034$, $\eta p2 = .104$. However, the Test x Group interaction was not statistically significant ($F(5.94, 110) = .625$, $p = .708$, $\eta p2 = .033$).
